# Effects of Shenlian extract on experimental atherosclerosis in ApoE-deficient mice based on ultrasound biomicroscopy

**DOI:** 10.1186/s12906-016-1449-6

**Published:** 2016-11-15

**Authors:** Yan Guo, Xu-Cen Liu, Ya-Jie Wang, Qi Li, Qing Yang, Xiao-Gang Weng, Ying Chen, Wei-Yan Cai, Xiao-Xi Kan, Xi Chen, He-Fei Huang, Xiao-Xin Zhu, Yu-Jie Li

**Affiliations:** Institute of Chinese Materia Medica, China Academy of Chinese Medical Sciences, No.16, Dongzhimen Nei Nanxiao Road, Dongcheng District, Beijing, 100700 China

**Keywords:** Atherosclerosis, Ultrasound biomicroscopy, Plaque, Hemodynamics, *Radix Salviae miltiorrhizae*, *Andrographis paniculata*

## Abstract

**Background:**

This study directly and dynamically investigated the effects of SL extract (i.e., a combination of *Radix Salviae miltiorrhizae* and *Andrographis paniculata* extract) on plaque progression in vivo by high resolution ultrasound biomicroscopy (UBM).

**Methods:**

An atherosclerosis model was established by placing a perivascular collar on the right common carotid artery in apolipoprotein E-deficient (ApoE^-/-^) mice. Thickness, plaque area and local blood flow were observed by UBM, pathological changes were observed by histochemical staining, and lipid levels were measured by respective commercially available kits.

**Results:**

Compared with the model group, the SL extract groups showed reduced wall thickness of the aortic arch (GC: *P* = 0.001, *P* = 0.002, and *P* < 0.001; LC: *P* < 0.001, *P* < 0.001, and *P* < 0.001; BC: *P* = 0.027, *P* = 0.017, and *P* = 0.003; respectively), which presented with retarded plaque progression of the cartoid artery with concordantly increased blood flow (*P* = 0.002 and *P* < 0.001) as visualized in vivo by UBM. Histological analysis confirmed the reduction of carotid atherosclerosis.

**Conclusions:**

The SL extract inhibited the formation of atherosclerotic plaques in an ApoE^-/-^ mice model by UBM analysis, and did so by effects that ameliorated local blood flow and improved blood lipid levels.

## Background

Atherosclerosis (As) is closely related to cardio-cerebrovascular disease which is a leading cause of mortality and disability in the world [1]. The pathogenesis of early atherosclerosis is thought to involve imbalanced lipid metabolism and low shear stress [2, 3]. Shear stress plays an important role in the development and distribution of atherosclerotic plaques, which are predominantly observed in the arterial branches and curvatures as a consequence of disturbed local blood flow [4, 5]. In addition, hyperlipidemia not only causes lipid deposition in the vascular intima, it also increases blood viscosity and reduces blood flow [6, 7].

In the pursuit of a rapid and representative atherosclerosis model, perivascular collar placement and a high-fat diet in ApoE-deficient (ApoE^-/-^) mice has been used in several studies. A high-fat diet promotes hyperlipidemia and constrictive collar results in vascular stenosis and changes the hemodynamic state of the carotid artery. In fact, the collar-induced carotid constriction model is very similar to the process that is involved in the formation of human carotid plaques [8, 9].

According to traditional Chinese medical theory, *Radix Salviae miltiorrhizae* can promote blood circulation and resolve blood stasis, *Andrographis paniculata* reveals significant clearance of endogenous heat toxins. Our previous studies have shown that Shenlian (SL) extract (i.e., a combination of *Radix Salviae miltiorrhizae* and *Andrographis paniculata* extract) could inhibit the formation of atherosclerotic plaques, reduce inflammatory biomarkers, regulate lipid metabolism and modulate blood viscosity in both rat and rabbit models [10–13]. Given this background, we therefore hypothesized that SL extract may affect several hemodynamic factors that are involved in atherosclerotic plaque formation, and yet direct evidence is lacking.

Ultrasound biomicroscopy (UBM) represents a potentially quick, non-invasive, real-time imaging approach, which can be used to obtain structural, functional and hemodynamic information [14]. This technology was widely used to detect atherosclerotic plaques in clinical studies, but its use was limited in small animal models because of the high heart rate and small vessel sizes that were found [15]. Therefore, and at least up to now, examination of atherosclerotic plaques has depended upon histopathology. However, in the past decade, and with the rapid development of high resolution UBM technology, high resolution imaging is possible down to 30 μm, which can measure the blood flow containing dynamic information in both speed and direction. UBM technology has been successfully used to observe plaque progress over time in other atherosclerotic models [16].

In the present study, we have established collar-induced and high-fat diet induced atherosclerotic models in ApoE^-/-^ mice. Further, we have used advanced high resolution UBM technology to further investigate the effects of SL extract on atherosclerosis with the intention of directly and dynamically observing changes in plaques and blood flow in vivo.

## Methods

### Chemicals and animals

Total Cholesterol (TC) kits, Triglyceride (TG) kits and high density lipoprotein cholesterol (HDL-C) kits were purchased from Jiancheng Bioengineering Institute (Nanjing, China). Hematoxylin-Eosin (H&E) staining kits were obtained from Solarbio Science and Technology Co., Ltd (Beijing, China).

Fifty male ApoE^-/-^ mice (aged 9 weeks old, on the C57BL/6 J genetic background) and ten male C57BL/6 J mice were purchased from HFK bioscience Co., Ltd (Beijing, China). Mice were maintained at an environmental temperature of 22 ± 2 °C and in a 12-h light-dark cycle controlled room. All animal experiments were approved by the local Laboratory Animal Ethics Committee of the Institute of Chinese *Materia Medica*, of the China Academy of Chinese Medical Sciences (Beijing, China; reference number 20142030) and performed in accordance with the guidelines for the care and use of laboratory animals.

### Preparation of the SL extract

*Radix Salviae miltiorrhizae* and *Andrographis paniculata* were obtained from Beijing tongrentang Co., Ltd (Beijing,China). The taxonomic authenticity was identified by Prof. Xirong He, the Institute of Chinese Materia Medica, of the China Academy of Chinese Medical Sciences (Beijing, China).

The SL extract [17] were composed of *Radix Salviae miltiorrhizae* extract and *Andrographis paniculata* extract at a ratio of 5:3. The *Radix Salviae miltiorrhizae* extract included two kinds of components, one was extracted with EtOH(ethanol) under percolation and then concentrated under reduced pressure, the other was then prepared by dilute EtOH soaking, and purified by macroporous resins SP825. The *Andrographis paniculata* extract was prepared by dilute EtOH soaking, and purified by macroporous resins SP825. The controllable components from SL extract were more than 60%, and TanshinoneIIA (3%), salvianolic acid B (38%) and andrographolide (20%) were detected in the SL extract by high performance liquid chromatography(HPLC).

### Surgery procedures and drug administration

The atherosclerotic model was established by perivascular constrictive silastic collars that were placed on the right common carotid arteries. ApoE^-/-^ mice were anesthetized by peritoneal injection of pentobarbital sodium (at a dose of 50 mg/kg). Then, the right common carotid artery was gently isolated. The constrictive collar (0.3 mm in inner diameter) was placed around the right common carotid artery, and with three surgical thread fixing collar. In C57BL/6J mice, the right common carotid artery was isolated without placing the constrictive collar.

One week after surgery, ApoE^-/-^ mice were randomly divided by weight into five groups of 10 animals for each group, which were then orally administered control or test treatments thus: the model group (0.5% carboxymethylcellulose sodium), the low-dose SL group (95 mg/kg), the medium-dose SL group (190 mg/kg), the high-dose SL group (380 mg/kg) and the atorvastatin (ATO) plus pioglitazone (PIO) group (4.6 mg/kg). Ten C57BL/6J mice belonged to the normal group and received oral administration as described for the model group. The normal group was fed a normal balanced diet, whereas mice in the other groups were fed a high-fat diet (containing 10% lard, 1% cholesterol, 10% egg yolk powder and a 79% basal diet). Diet and water were provided ad libitum. The doses and proportion of SL extract were mainly determined by the early stage of the research and the clinical dosage of *Radix Salviae miltiorrhizae* and *Andrographis paniculata*. According to the extraction percentage of crude drugs, the doses of 95, 190, 380 mg/kg were the equivalent dose, 2 times and 4 times of the clinical dosage.

At the end of the experimental period (i.e., 8 weeks), high resolution UBM measurements were performed from randomly selected sets of five mice per group. After ultrasound detection and an overnight fast, mice were anesthetized with pentobarbital sodium (at 50 mg/kg). Blood was taken by removing an eyeball and serum specimens were obtained by centrifugation of the collected blood (i.e., at 3000 rpm for 15 min at 4 °C). The right common carotid arteries were removed expeditiously and stored in a −80 °C freezer.

### UBM

Before examining the animals by UBM, each group of mice were anesthetized by peritoneal injection of pentobarbital sodium (at a dose of 50 mg/kg). The hair of the chest and neck was shaved away carefully. Then, mice were laid in the supine position on a platform that could keep the body temperature at 36–38 °C and the heart rate was then monitored. Ultrasound transmission gel was applied to the chest as an acoustic coupling medium. UBM was measured with a high resolution in vivo micro-imaging system (Vevo 2100 system, Visualsonics, Toronto, Canada). The imaging system was equipped with an MS550D transducer operating at 40 MHz with an axial resolution of 40 μm for all examinations.

First, with the device on B-mode, a long axis view was used to visualize the aortic arch. Plaque and wall thicknesses of the aortic arch were observed on the image. The measured wall thickness of the aortic arch included the greater curvature (GC), the lesser curvature (LC) and the origin of the brachiocephalic artery (BC), which were known to be sites of atherosclerotic plaques. Secondly, on B-mode, a long axis view was taken to visualize the plaque of the right common carotid arteries. On the basis of the position of the constrictive collar, the distal and proximal carotid arteries were observed respectively. In particular, the proximal carotid artery was highly predisposed to atherosclerotic plaques. Then, a short axis view was used to visualize the largest plaque site of the proximal carotid artery, and the plaque area was measured on the short axis image. Third, on the PW Doppler Mode, the blood flow of the aortic arch and the distal and proximal carotid arteries were measured respectively using the color Doppler, which converted Doppler sounds into colors that were overlaid on the image of the blood vessel to represent both the speed and direction of blood flow through the vessel. Moreover, the region of blood flow was indirectly reflected by the physical location of the colors.

### Serum lipid measurements

The serological levels of TC, TG and HDL in serum were measured using a commercially available kit. LDL levels were calculated using the Friedewald formula, thus: LDL = TC-HDL-TG/2.2 (mmol/L).

### Histopathological examination of the right common carotid artery

The right common carotid arteries were embedded in an optical coherence tomography (OCT) compound. Serial cryosections (i.e., at 6 μm each) were cut sequentially at 50 μm intervals along samples of the carotid artery and stained routinely with H&E. Pathological changes were observed by microscopy (BX51, Olympus Company, Tokyo, Japan).

### Statistical analysis

All reported data were presented as the mean ± SD and were processed using the statistical analysis software SPSS version 17.0 (SPSS Inc., Chicago, IL, USA). Statistical analyses were performed by one-way analysis of variance (ANOVA), and an LSD *post hoc* test was used to show differences for each pair of factor levels. A *p* value < 0.05 was considered statistically significant.

## Results

### Effect of SL extract on the serum levels of TC, TG, HDL and LDL in ApoE^-/-^ mice

**Fig. 1:**
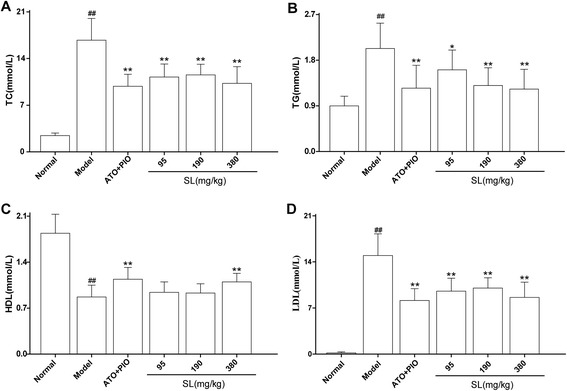
Effect of SL extracts on the serum levels of TC (**a**), TG (**b**), HDL (**c**) and LDL (**d**) levels in ApoE^-/-^ mice. The results are expressed as mean ± SD of 15 animals per group, ^##^ *P* < 0.01: significantly different from the normal group and ^*^ *P* < 0.05, ^**^ *P* < 0.01: significantly difference as compared with the model group

As shown in Fig. 1, in comparison with the normal group, serological levels of TC, TG and LDL were increased significantly and HDL was reduced significantly in the model group (*P* < 0.001). However, the low, medium and high-dose SL groups showed significantly reduced levels of TC, TG and LDL as compared with the model group (TC: *P* < 0.001, *P* < 0.001 and *P* < 0.001; TG: *P* = 0.015, *P* < 0.001 and *P* < 0.001; LDL: *P* < 0.001, *P* < 0.001 and *P* < 0.001; respectively). By contrast, increased HDL levels were detected in the high-dose SL group (*P* = 0.007).

### Morphological changes of the right common carotid artery

**Fig. 2:**
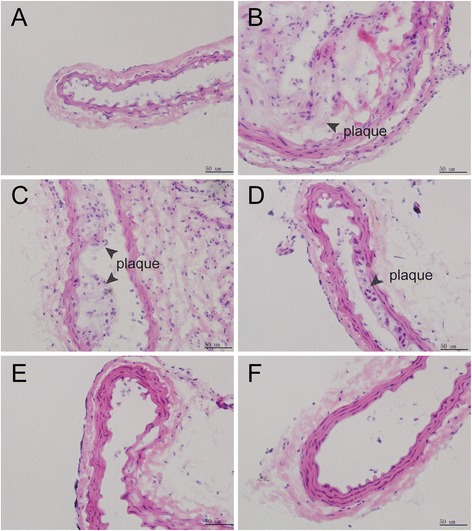
Hematoxylin and eosin stained cross-sections of the proximal carotid artery in ApoE^-/-^ mice (magnification, ×400). **a** normal group, **b** model group, **c** low-dose SL group, **d** medium-dose SL group, **e** high-dose SL group, and **f** ATO plus PIO group. The *black* bar on the bottom of the images represents a scale of 50 μm

As shown in Fig. 2, the structure of the vascular intima, media and adventitia were neat and displayed full integrity, and no histopathological changes were observed in the normal group. However, in the model group, the structure of the vascular intima and media were damaged. By contrast, the vascular intima became thicker and fewer smooth cells appeared in the vascular media. Moreover, the carotid arterial lumen was densely packed with atherosclerotic plaques that contained a large number of foam cells, lipid droplets and inflammatory cells. As compared with the model group, the SL treatment groups showed obvious and dose-dependent suppression of those changes and fewer atherosclerotic plaques were observed.

### Alterations of wall thickness of aortic arch measured by UBM

**Fig. 3:**
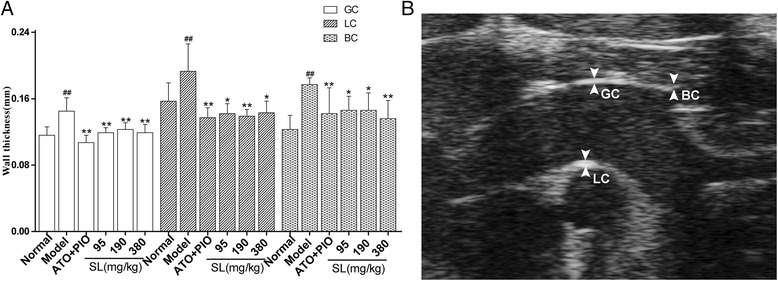
UBM of the aortic arch in ApoE^-/-^ mice. **a** Wall thickness of the aortic arch was observed at the greater curvature (GC), the lesser curvature (LC) and the origin of the brachiocephalic artery (BC). Each value is expressed as the mean ± SD of five animals per group, ^##^ *P* < 0.01: significantly different from the normal group and ^*^ *P* < 0.05, ^**^ *P* < 0.01: significantly difference as compared with the model group. **b** The position of the aortic arch illustrates that measurements were observed by UBM

Wall thickness of the aortic arch was measured by UBM, As shown in Fig. 3, and as compared with the normal group, the wall thickness of the GC, LC and BC were significantly increased in the model group (*P* = 0.001, *P* = 0.004, and *P* < 0.001; respectively). However, the low, medium and high-dose SL groups showed significantly reduced wall thicknesses of the GC, LC and BC as compared with the model group(GC: *P* = 0.001, *P* = 0.002, *P* < 0.001; LC: *P* < 0.001, *P* < 0.001, *P* < 0.001; BC: *P* = 0.027, *P* = 0.017, and *P* = 0.003; respectively).

### Atherosclerotic plaque observation and measurement of the distal and proximal carotid artery by UBM

**Fig. 4:**
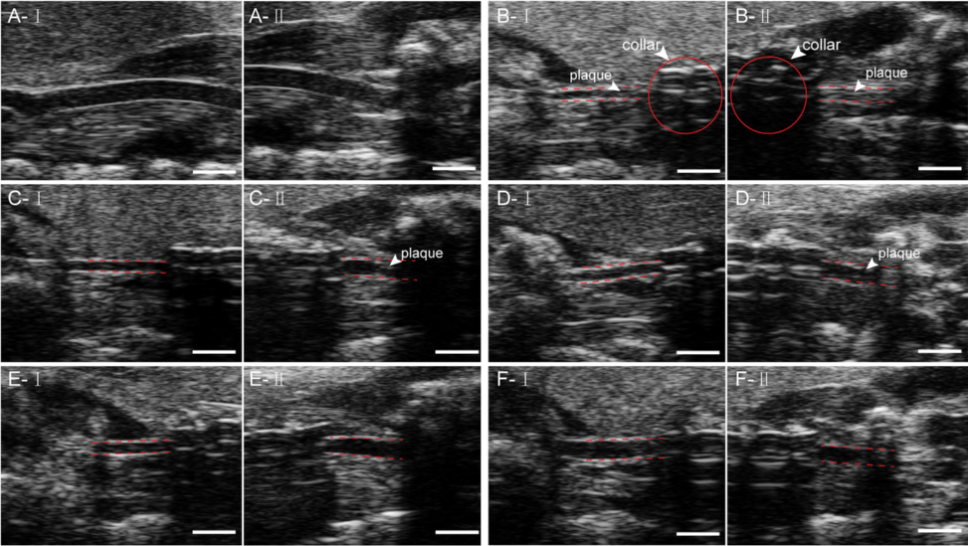
Ultrasound long-axis views of the right common carotid arteries in ApoE^-/-^ mice. **a** normal group, **b** model group, **c** low-dose SL group, **d** medium-dose SL group, **e** high-dose SL group, and **f** ATO plus PIO group. Each group numberIand II represent the distal and proximal carotid artery on the basis of the position of the constrictive collar. The white bar on the bottom of the images represents 1 mm

On the basis of the positon of the constrictive collar, atherosclerotic plaques in the distal and proximal areas of the carotid artery were clearly and respectively visualized by UBM in long axis views. As shown in Fig. 4, the carotid arterial lumen was densely packed with atherosclerotic plaques in the model group. This was particularly evident in the proximal area of the carotid artery, where the lumen was almost completely blocked by plaque formation, and the vessel walls were unclear. However, in the SL treatment groups, no plaque formation was detected in the distal zone of the carotid artery, and presence of atherosclerotic plaques in the proximal carotid artery were less than were found in the model group, in which the lumen became clearer.

**Fig. 5:**
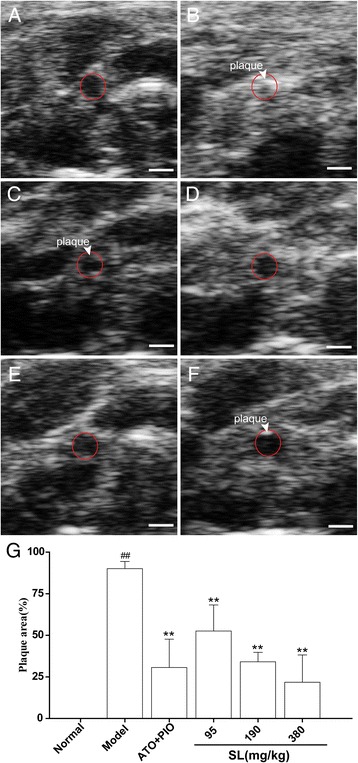
Ultrasound shot-axis views of the right common carotid arteries in ApoE^-/-^ mice. **a** normal group, **b** model group, **c** low-dose SL group, **d** medium-dose SL group, **e** high-dose SL group, and **f** ATO plus PIO group. The red lines identify the borders of the carotid arteries. The *white bar* on the bottom of the images represents a scale of 0.5 mm. **g** Quantitative analysis of the UBM for the plaque area. The results are presented as mean ± SD of five mice per group, ^##^ *P* < 0.01: significantly different from the normal group and ^**^ *P* < 0.01: significantly different from the model group

The maximum plaque area in the proximal part of the carotid artery was measured by UBM in short axis views. As shown in Fig. 5, the cross-sectional lumen of the carotid artery was narrowed by the presence of plaques at a rate of 90.1 ± 4.3% in the model group. As compared with the model group, the plaque area in the low, medium and high-dose SL groups were significantly reduced (52.6 ± 15.7%, 34.1 ± 5.7%, 21.8 ± 16.4%; *P* < 0.001, *P* < 0.001, and *P* < 0.001; respectively) in a dose-dependent manner.

### Effect of SL extract on the blood flow of distal and proximal carotid arteries by UBM

**Fig. 6:**
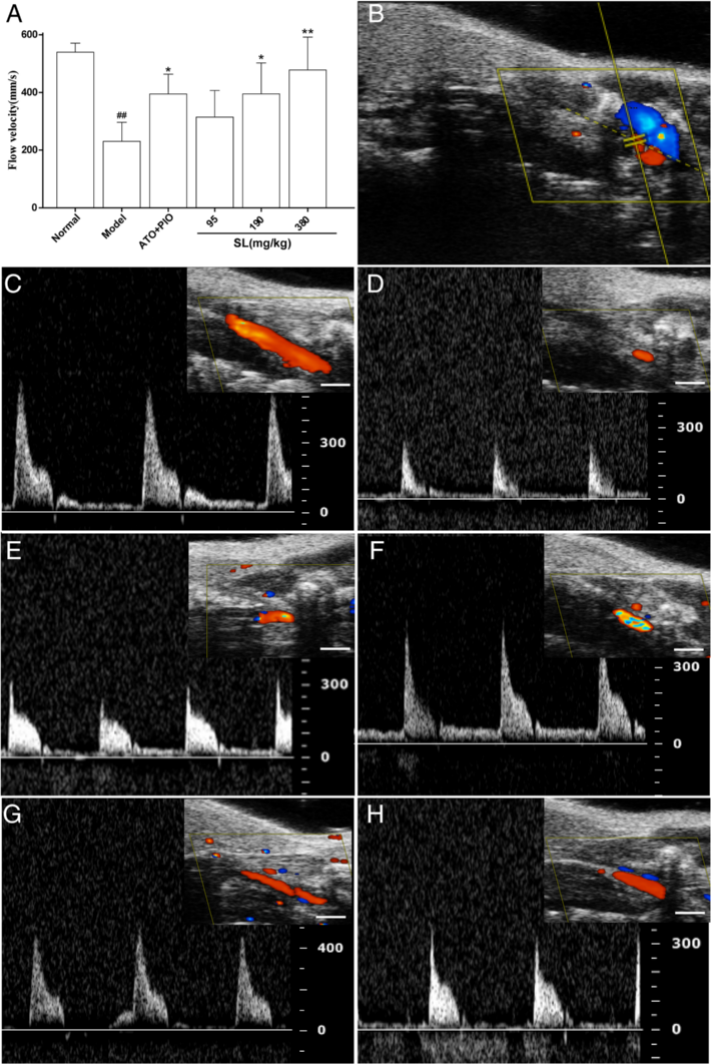
UBM of blood flow on the distal carotid artery in ApoE^-/-^ mice. **a** Quantitative analysis of the blood flow velocity by UBM. The results are presented as mean ± SD of five mice per group, ^##^ *P* < 0.01: significantly different from the normal group and^*^ *P* < 0.05, ^**^ *P* < 0.01: significantly different from the model group. **b** The image shows the position of the color Doppler sample volume. **c** normal group, **d** model group, **e** low-dose SL group, **f** medium-dose SL group, **g** high-dose SL group, and **h** ATO plus PIO group. The white bar on the bottom of the images represents a scale of 1 mm

The blood flow of the carotid artery was changed because of elevated levels of blood lipids and the presence of atherosclerotic plaques. On the basis of the position of the constrictive collar, blood flow in the distal and proximal areas of the carotid artery was clearly and respectively visualized by color Doppler UBM. As shown in Fig. 6, the region and velocity of the blood flow in the proximal carotid artery was small and low in the model group. In addition, as compared with the model group, the region and velocity of blood flow in the SL treatment groups were all increased, and the medium and high-dose SL groups resulted in a significant increase in blood flow velocity as compared with the model group (*P* = 0.020 and *P* = 0.002).

**Fig. 7:**
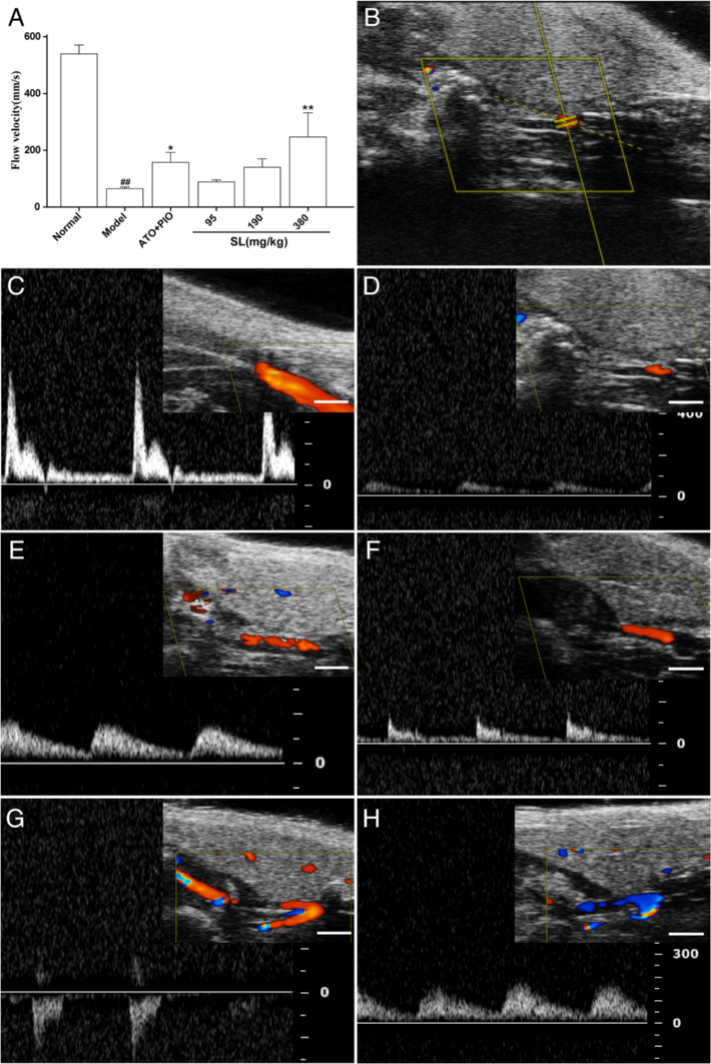
UBM of blood flow on the proximal carotid artery in ApoE^-/-^ mice. **a** Quantitative analysis of the blood flow velocity by UBM. The results are presented as mean ± SD of five mice per group, ^##^ *P* < 0.01: significantly different from the normal group and^*^ *P* < 0.05, ^**^ *P* < 0.01: significantly different from the model group. **b** The image shows the position of the color Doppler sample volume. **c** normal group, **d** model group, **e** low-dose SL group, **f** medium-dose SL group, **g** high-dose SL group, and **h** ATO plus PIO group. The white bar on the bottom of the images represents a scale of 1 mm

As shown in Fig. 7, the result of blood flow measurements in the distal carotid artery was similar to that found for the proximal carotid. The high-dose SL group also showed markedly increased blood flow velocity as compared the model group (*P* < 0.001).

## Discussion

Atherosclerosis is a common and complex pathological process. Hyperlipidemia and low shear stress contribute to the development of atherosclerosis. In the present study, we successfully established a rapid carotid atherosclerosis model by using a high-fat diet and perivascular collar placement in ApoE^-/-^ mice (with a post-surgical survival rate of 100%). A high-fat diet increases blood viscosity and placement of a constrictive collar results in site-controlled atherogenesis, and did so based on changing the hemodynamic state of the carotid artery. Therefore, this model is highly physiologically relevant to the human condition seen in atherosclerosis development, and the model was deemed suitable for our research objectives.

The SL extract was comprised of *Radix Salviae miltiorrhizae* and *Andrographis paniculata*, which were highly compatible in our model. The major effective components of the extracts included Salvianolic acid B, TanshinoneIIA and Andrographolide, which are considered to have biological effects in vascular disease. Previous studies have reported that Salvianolic acid B inhibits platelet adhesion to collagen, and does so by interfering with the function of collagen receptor α2β1 [18], regulating lipid metabolism by inhibiting CD36 expression [19] and via oxidative modification of low density lipoprotein (LDL). In addition, TanshinoneIIA can promote blood flow by inhibiting platelet aggregation [20] and activation [21], and reducing thrombus formation [22]. Andrographolide has a significant anti-inflammatory effect by inhibiting the activation of the transcription factor NF-κB [23, 24]. In the current study, we observed that SL extract regulated lipid metabolism by reducing the levels of TC, TG and LDL, and increasing HDL-C in the serum as compared with the model group. Furthermore, SL extract inhibited formation of carotid atherosclerotic plaques based on histopathological examination. These results coincided with our previous studies. However, the highlight of this study was the use of high-resolution UBM to directly and correctly observe carotid atherosclerosis and measure changes in local blood flow in vivo.

High resolution UBM technology has been widely used to detect atherosclerotic plaques in animal models [25, 26]. Compared with the traditional study of atherosclerotic plaques, there are several unique advantages inherent in UBM approaches. First, UBM is non-invasive [27, 28] on atherosclerotic plaques in vivo, and yet traditional studies require sacrificing animals to gather information on plaques. Secondly, UBM is very intuitive [29] in observing the existence of plaques, which includes information on their location, and their size. Traditional research needs histological measurements, which is a complex process. Thirdly, the results of UBM on plaque examination are reliable and precise. Previous studies [14] had demonstrated that UBM and histopathology data revealed positive correlations for plaque area, intima-medial thickness, remodeling index and eccentric index. Fourth, the information gained by UBM is quite comprehensive. It includes changes in structure, function and hemorheology [30–32]. Finally, UBM approaches can be employed as a real-time imaging tool to study plaque progression. Previous research had reported that temporal changes in plaque area were measured by UBM in a mouse model [16]. However, few studies have applied UBM to the study of carotid plaques and observed subsequent effects of drug therapy. Hence, based on our previous studies and the advantages of UBM, we applied high resolution UBM to observe the effects of SL extract on carotid atherosclerosis in ApoE^-/-^ mice.

In the present study, three aspects were observed when using UBM on carotid atherosclerosis. First and foremost, we measured the wall thickness of GC, LC and BC in the aortic arch where the spontaneous atherosclerotic plaque was preferentially located. SL treatment groups showed significantly reduced wall thickness for GC, LC and BC as compared with the model group. This result suggested that the SL extract could inhibit the progression of spontaneous atherosclerotic plaques in ApoE^-/-^ mice. Moreover, we measured the plaque area of the right common carotid artery, which was constricted by the placement of the collar. On B-mode, no plaque was detected in the distal area of the carotid artery as compared with the model group. Additonally, the atherosclerotic plaque of the proximal carotid artery was less than the model group, and the lumen became clearer in the long axis views. The plaque area in the SL treatment groups were significantly reduced in a dose-dependent manner in short axis views. This suggested that the SL extract could reduce collar-induced carotid atherosclerotic plaques in ApoE^-/-^ mice, and this was highly correlated to the histological examination. Finally, we measured the blood flow [33] in the aortic arch and the distal and proximal area of the carotid artery respectively. On PW Doppler mode, the SL treatment groups showed significantly increased blood flow in the distal and proximal areas of the carotid artery as compared with the model group, but this observation was not statistically significant in the aortic arch (data not shown). These results indicated that the SL extract had a more significant impact upon locally abnormal blood flow in the impaired regions.

## Conclusions

Based on the high-fat diet and perivascular collar placement on the carotid artery in the ApoE^-/-^ mice model, we directly and dynamically demonstrated that the SL extract inhibited formation of atherosclerotic plaques in the carotid artery and spontaneous formation in the aortic arch using advanced high resolution UBM. Moreover, we did so by effects that were dependent on direct amelioration of local blood flow of the collar-induced constrictive carotid artery and improvement of blood lipid levels.

## Abbreviations

ApoE^-/-^: Apolipoprotein E-deficient
As: Atherosclerosis
ATO: Atorvastatin
H&E: Hematoxylin and eosin
HDL-C: High density lipoprotein cholesterol
HPLC: High performance liquid chromatography
LDL: Low density lipoprotein
OCT: Optical coherence tomography
PIO: Pioglitazone
SL extract: Shenlian extract
TC: Total cholesterol
TG: Triglyceride
UBM: Ultrasound biomicroscopy
